# Chronic hepatitis B virus infection drives changes in systemic immune activation profile in patients coinfected with *Plasmodium vivax* malaria

**DOI:** 10.1371/journal.pntd.0007535

**Published:** 2019-06-24

**Authors:** Luís A. B. Cruz, Marina O. A. Moraes, Matheus R. Queiroga-Barros, Kiyoshi F. Fukutani, Manoel Barral-Netto, Bruno B. Andrade

**Affiliations:** 1 Instituto Gonçalo Moniz, Fundação Oswaldo Cruz (FIOCRUZ), Salvador, Brazil; 2 Faculdade de Tecnologia e Ciências (FTC), Salvador, Brazil; 3 Multinational Organization Network Sponsoring Translational and Epidemiological Research (MONSTER) Initiative, Fundação José Silveira, Salvador, Brazil; 4 Universidade Salvador (UNIFACS), Laureate Universities, Salvador, Brazil; 5 Universidade Federal da Bahia, Faculdade de Medicina, Salvador, Brazil; 6 Instituto Nacional de Ciência e Tecnologia, Instituto de Investigação em Imunologia, São Paulo, Brazil; 7 Escola Bahiana de Medicina e Saúde Pública (EBMSP), Salvador, Brazil; Universidade Federal de Minas Gerais, BRAZIL

## Abstract

**Background:**

*Plasmodium vivax* and Hepatitis B virus (HBV) are globally outspread in similar geographic regions. The concurrence of both infections and its association with some degree of protection against symptomatic and/or severe vivax malaria has been already described. Nevertheless, data on how host response to both pathogens undermines the natural progression of the malarial infection are scarce. Here, a large cohort of vivax malaria and HBV patients is retrospectively analyzed in an attempt to depict how inflammatory characteristics could be potentially related to the protection to severe malaria in coinfection.

**Methods:**

A retrospective analysis of a databank containing 601 individuals from the Brazilian Amazon, including 179 symptomatic *P*. *vivax* monoinfected patients, 145 individuals with asymptomatic *P*. *vivax* monoinfection, 28 *P*. *vivax*-HBV coinfected patients, 29 HBV monoinfected subjects and 165 healthy controls, was performed. Data on plasma levels of multiple chemokines, cytokines, acute phase proteins, hepatic enzymes, bilirubin and creatinine were analyzed to describe and compare biochemical profiles associated to each type of infection.

**Results:**

Coinfected individuals predominantly presented asymptomatic malaria, referred increased number of previous malaria episodes than symptomatic vivax-monoinfected patients, and were predominantly younger than asymptomatic vivax-monoinfected individuals. Coinfection was hallmarked by substantially elevated concentrations of interleukin (IL)-10 and heightened levels of C-C motif chemokine ligand (CCL)2. Correlation matrices showed that coinfected individuals presented a distinct biomarker profile when compared to asymptomatic or symptomatic *P*. *vivax* patients, or HBV-monoinfected individuals. Parasitemia could distinguish coinfected from symptomatic or asymptomatic *P*. vivax-monoinfected patients. HBV viremia was associated to distinct inflammatory profiles in HBV-monoinfected and coinfected patients.

**Conclusion:**

The findings demonstrate a distinct inflammatory profile in coinfected patients, with characteristics associated with immune responses to both pathogens. These host responses to *P*. *vivax* and HBV, in conjunction, could be potentially associated, if not mainly responsible, for the protection against symptomatic vivax malaria.

## Author summary

The determinants of the diverse clinical presentations of *Plasmodium vivax* malaria are not completely understood. Previous studies have reported that *P*. vivax-HBV coinfection is associated with increased odds of presenting with asymptomatic malaria, but little is known about the immune mechanisms driving such association. To illuminate host pathways associated with protection against malaria, we analyzed multiple cytokines, chemokines and acute phase proteins in groups of patients from the Brazilian Amazon with different presentations of vivax malaria monoinfection, HBV monoinfection, and *P*. *vivax*-HBV coinfection. The results indicate that coinfection is hallmarked by a conjunction of immune responses, related to each one of the monoinfections, that results in a balanced inflammation associated with clinical immunity and absence of symptoms. In biological terms, the readouts are that the combined responses to each pathogen would induce a distinct profile of systemic immune activation, with the hallmarked activity of IL-10, a classical immunoregulatory cytokine, in confluence mainly with CCL2 and IL-4 activity. These multiple pathways would prevent the unbalanced proinflammatory activity associated with symptomatic and/or severe vivax malaria. Moreover, these findings highlight the importance of the immune system in driving disease presentation, raise discussion of immunotherapy in vivax malaria, and how these approaches have the potential to influence clinical outcomes.

## Introduction

Malaria still rises major concerns in public health worldwide. The burden caused by the disease is noticeable, as it leads to more than 200 million cases and billions of dollars invested each year [1]. Despite all the investments and increased interest in the pursuit of new interventions [1,2], there was an elevation in the number of estimated cases in the successive years of 2016 and 2017 [1]. Moreover, *P*. *vivax*, which is the most widespread of the five main species of *Plasmodium* [1,3,4], have been increasingly associated with severe disease presentations and mortality [1,5–8].

Hepatitis B virus (HBV) infections are no less of a problem, with more than 250 million chronic cases estimated in 2015 [9]. Incidence of HBV has been reduced since the introduction of the vaccine, however approximately 815,000 deaths were accountable to HBV infections and its chronic complications in 2016 [10]. Both hepatitis B and vivax malaria are mainly outspread in tropical countries [1,9], and there is overlapping occurrence of these diseases [11].

HBV-associated tissue damage is described to be directly related to the host inflammatory response against infection [12,13]. The immune responses in chronic HBV infections are characterized by decreased T-cell proliferation potential and exhaustion [14–18]. Although not completely understood, these events are thought to be related to a higher release of HBsAg particles (hence, viral load) in the circulation, expression of co-inhibitory receptors, and production of IL-10 [14–21]. In vivax malaria, intensity of immune activation is associated with worse clinical outcomes [5,6,22–24]. On the converse, cases of asymptomatic *P*. *vivax* infection are hallmarked by a less pronounced pro-inflammatory response, with increased IL-10 levels in peripheral blood [6,11,22]. Thus, at first glance, both HBV and *P*. *vivax* infections seem to drive similar profiles of systemic inflammation in distinct clinical settings. Nevertheless, no previous study has performed a detailed characterization of systemic immune activation profile in HBV-malaria comorbidity.

Another similarity between HBV and malarial biology is the participation of the liver as a key organ part of the immunopathogenesis in both infections [4,7]. Notably, severe vivax malaria is associated with remarkable hepatic involvement [5–7,22,25], whereas tissue damage is determinant for the presentation of cirrhosis and hepatocellular carcinoma in chronic HBV infections [12,16,26]. Counterintuitively, HBV infection has been shown to lead to a distinct systemic inflammatory response in *Plasmodium* infections, resulting in increasing odds for asymptomatic malaria [11]. The present study expands the current knowledge as it examines in detail a rich interplay of cytokines, chemokines and acute phase proteins in a large number of patients infected with *P*. *vivax*, HBV or both. These analyzes demonstrate the nuances of different inflammatory responses in confluence, which culminates in an intense but balanced immune response to both pathogens, with key participation of relevant biomarkers as TNF-α, IL-4, IL-10 and CCL2.

## Methods

### Ethics statement

Written informed consent was obtained from all participants or their legally responsible guardians, and all clinical investigations were conducted according to the principles expressed in the Declaration of Helsinki. The project was approved by the institutional review board of the Faculdade de Medicina, Faculdade São Lucas, Rondônia, Brazil, where the study was performed.

### Study design

The present study is based on analyses performed retrospectively in databank containing immunological, clinical and epidemiological data from 601 subjects, including uninfected controls, recruited between 2006 and 2007 from the state of Rondônia, in the Brazilian Amazon. Multiple investigations have been reported from the project which this study is a part of [5,6,11,22–25,27–30]. Patient investigation included both active case detection in the municipalities of Buritis and Demarcação (Rondônia, Brazil) and passive case detection from individuals who sought care at Brazilian National Foundation of Health (FUNASA) diagnostic centers or at the municipal hospital in Buritis (Rondônia, Brazil).

Malaria diagnosis was conducted through microscopic examination of thick smears and nested polymerase chain reaction (PCR) evaluation in whole blood samples (20mL), with control for cross-contamination, performed at the Instituto Gonçalo Moniz (Fiocruz-BA), Salvador, Bahia, Brazil, as previously reported [5,6,22–24]. Individuals who tested positive through PCR evaluation and persisted without the presentation of fever (axillary temperature >37.8°C) and/or sweating, chills, jaundice, myalgia, arthralgia, asthenia, nausea, and emesis for 30 days were considered asymptomatic. Patients, which parasitological tests were positive, presenting any symptom listed above, were considered symptomatic. HBV diagnosis was conducted employing the AXSYM automatic ELISA system (Abbott, Wiesbaden, Germany), HBSAg, HBeAg, total anti-HBS, total anti-HBc, anti-HBc IgM and anti-HBe IgG were screened, according to the most updated protocols published by the Brazilian Ministry of Health at the time of study enrollment, and no acute HBV infection was detected (HBSAg+, anti-HBS-, anti-HBc IgM+). All the measurements were performed right at the study enrollment and diagnosis of malarial and/or HBV infection, meaning that the collections were performed before the initiation of antimalarial or HBV-specific therapy. Information regarding the number of previous malaria episodes and years that the patients resided in the area at the time of study enrollment were obtained directly from the patients in the interview part of the medical examination. For the present study patients with both symptomatic (n = 179) and asymptomatic (n = 145) *P*. *vivax* monoinfection, ongoing HBV infection (n = 29), concurrent *P*. *vivax* and HBV infections (n = 28) and healthy controls (n = 165, from which 152 had all the epidemiological data available) were included. The exclusion criteria for the present study were: patients with documented *P*. *falciparum* or HIV infections, tuberculosis, cancer, or use of immunosuppressant drugs. For the analyses of biochemical markers, patients presenting *P*. *vivax* monoinfection who were previously infected by HBV were excluded, in order to avoid interferences on the inflammatory profile. In addition, for part of these analyses, *P*. *vivax* monoinfected individuals, independently of clinical status (symptomatic or asymptomatic), were considered as a single group (n = 268), to compare and attest if the factors involved in the clinical presentation from coinfected subjects would also differ *P*. *vivax*-HBV coinfection from the *P*. *vivax* monoinfection overall. Clinical, demographic and epidemiological characteristics of the participants included in the current study are described in Tables 1 and 2 and S1.

**Table 1:** Characteristics of the study subpopulations.

	*P*. *vivax* malaria patients			HBV patients	healthy controls
Variables	asymptomatic	symptomatic	HBV coinfected		
**N**	145*	179	28	29	165*
**Male**–no. **(%)**	63 (45.65)	100 (55.87)	16 (57.14)	16 (55.17)	72 (47.37)
**Age**–yrs. ^***a***^					
Median	43	32	30.5	38	39
Interquartile interval	34–51.25	24–47	22.5–45.75	22.5–45.75	25–51 _**θ**_
*Previous malaria episodes ^***b***^					
Median	17	5	18	12	13
Interquartile interval	13–20	1–11	10.25–25	10.25–14	10–18 _**θ**_
**Previous HBV**–no. **(%)**	18 (13.04)	38 (21.23)	___	___	___
**Years resident in the area–**no. **(%)** ^***c***^					
<2yrs.	25 (18.11)	52 (29.05)	2 (7.14)	3 (10.35)	39 (25.66)
3-10yrs.	14 (10.15)	46 (25.70)	3 (10.72)	4 (13.79)	16 (10.52)
>10yrs.	99 (71.74)	81 (45.25)	23 (82.14)	22 (75.86)	97 (63.82)
**Parasitemia (count/μL)** ^***a***^^,^^***d***^				__	__
Median	0	6324	753		
Interquartile interval	0–32	913.5–60,623	444.3–4,262		

Frequency data were compared using the chi-square test or the Fisher’s exact test. Continuous variables were compared using the Mann-Whitney U test or the Kruskal-Wallis test with Dunn’s multiple comparisons test.

*From 165 and 145 subjects recruited as endemic controls and asymptomatic *P*. *vivax* patients, respectively, only 152 and 138 had all the epidemiological data available. One symptomatic vivax malaria and 17 HBV patients could not recall the number of previous malaria episodes. HBSAg, HBeAg, total anti-HBS, total anti-HBc, anti-HBc IgM and anti-HBe IgG were screened, and no acute HBV infection was detected.

^***a***^ *=* Differences were significant between asymptomatic against other *P*. *vivax* infected patients.

^***b***^ = Differences were significant between symptomatic against other *P*. *vivax* infected patients.

^***c***^ = Differences were significant between the proportions.

^***d***^ = Differences between symptomatic vivax malaria and coinfected were significant on Mann-Whitney U test analysis.

_**θ**_ = Differences were significant between controls and both symptomatic or asymptomatic vivax malaria patients.

**Table 2:** Biochemical evaluation of the study subpopulations.

	*P*. *vivax* malaria patients			HBV patients	P value
Variables	asymptomatic	symptomatic	HBV coinfected		
**N**	127	141	28	29	__
**TNFα**– pg/mL	1.8 (0–9.1)	38.6 (12.55–81.95)	22.7 (5.85–33.13)	43.1 (28.45–56.45)	<0.0001
**IFN-γ**– pg/mL	75.7 (24.0–165.0)	100 (33.2–324.8)	429.2 (262–560)	321 (100.4–570.8)	<0.0001
**IL-1β**– pg/mL	3.68 (2.53–7.66)	12 (5.68–28.4)	5.64 (5.29–6.14)	4.75 (1.18–15.2)	<0.0001
**IL-4**– pg/mL	20.68 (11.38–34.41)	30.07 (16.54–115.5)	46.89 (25.94–122.4)	1.83 (0.64–9.85)	<0.0001
**IL-6**– pg/mL	10.2 (0–20.4)	61.2 (21.9–121.5)	29.4 (20.4–47.75)	35.5 (22.0–46.15)	<0.0001
**IL-8**– pg/mL	3.16 (2.17–6.41)	26.75 (5.94–117.7)	2.59 (1.79–4.53)	11.12 (7.03–17.66)	<0.0001
**IL-10**– pg/mL	63.0 (32.8–94.0)	12.5 (6.47–45.4)	497.8 (221.4–823.7)	23 (15.8–33.95)	<0.0001
**IL-12p70**– pg/mL	10.82 (7.27–17.57)	21.05 (10.45–32.65)	5.35 (4.68–7.25)	3.88 (1.82–12.31)	<0.0001
**CRP**–mg/L	7.8 (4.5–10.3)	15.5 (7.8–33.75)	7.0 (4.55–10.45)	4.8 (4.0–6.75)	<0.0001
**CCL2**– ng/mL	87.81 (17.22–168.5)	60.3 (18.3–153.8)	188.7 (136.4–325.7)	96.81 (46.41–167.1)	<0.0001
**CCL5**– μg/mL	23,473 (12,279–36,450)	25,266 (16,193–77,783)	29,128 (14,051–89,644)	32,650 (17,889–44,330)	0.0055
**CXCL9**– ng/mL	473.8 (247.8–761.5)	2,386 (470.8–10,678)	724.1 (174–1,657)	583.7 (220.1–1,467)	<0.0001
**CXCL10**– pg/mL	13.53 (9.09–21.98)	81.7 (26.27–421.2)	64.02 (110.8–142.5)	126.0 (78.23–162.1)	<0.0001
**Fibrinogen**–mg/dL	293.4 (204.5–345.9)	384.5 (234.5–495.5)	338.0 (247–416.8)	203.5 (176.2–240.5)	<0.0001
**AST**–U/L	49.5 (34.5–77.5)	180.0 (87.62–644.7)	65.45 (32.75–103.6)	45.0 (34.15–55.5)	<0.0001
**ALT**–U/L	39.5 (29.7–65.0)	190.3 (128.9–496.5)	42.75 (23.05–75.98)	37.4 (33.45–43.45)	<0.0001
**Total Bilirubin**–mg/dL	0.70 (0.49–1.00)	1.20 (0.80–2.00)	1.20 (0.80–1.60)	0.80 (0.54–1.15)	<0.0001
**Direct Bilirubin**–mg/dL	0.40 (0.20–0.70)	0.50 (0.30–0.85)	0.54 (0.30–0.80)	0.40 (0.30–0.50)	0.1086
**Indirect Bilirubin**–mg/dL	0.24 (0.20–0.30)	0.70 (0.47–1.30)	0.38 (0.26–0.72)	0.40 (0.27–0.62)	<0.0001
**Creatinine–**mg/dL	1.20 (0.97–1.29)	1.27 (1.18–1.38)	0.98 (0.68–1.28)	1.2 (0.83–1.46)	<0.0001

Data represent interquartile range. Variables were compared using the Kruskall-Wallis test with Dunn’s multiple comparisons test.

### Laboratory measurements

Plasma levels of cytokines IL1-β, IL-4, IL-6, IL-10, IL-12p70, IFN-γ, tumor necrosis factor (TNF)-α, C-C motif chemokine ligand (CCL)2, CCL5, C-X-C motif chemokine ligand (CXCL)9, and CXCL10 were measured using the Cytometric Bead Array—CBA (BD Biosciences Pharmingen, San Diego, CA, USA), according to the manufacturer’s protocol. The measurements of aspartate amino-transferase (AST), alanine amino-transferase (ALT), total bilirubin, direct bilirubin, creatinine, fibrinogen and C-reactive protein (CRP) were performed at the Pharmacy School of the Federal University of Bahia and at the clinical laboratory of Faculdade São Lucas.

### Statistical analysis

The median values with interquartile ranges (IQR) were used as measures of central tendency and dispersion. Chi-square test was used to compare frequencies between the study groups. Continuous variables were compared between the study groups using the Mann-Whitney *U* test (2-group comparisons), or the Kruskall-Wallis test with Dunn’s multiple comparisons ad hoc test (between 3 or more groups). Hierarchical cluster analyzes were performed using the Ward’s method with bootstrap (100X). Spearman tests were performed to analyze correlations and to build the correlation matrices, which assessed markers in each study group. Only correlations with Spearman rank (r) values above 0.6 were plotted in the matrices. A p-value below 0.05 after adjustment for multiple measurements (false discovery rate of 1%) was considered statistically significant. The statistical analyzes were performed using Graphpad Prism 7.0 (GraphPad Software Inc., San Diego, CA, USA), and JMP 12.0 (SAS, Cary, NC, USA).

## Results

### Characteristics of the study participants

The baseline characteristics of the study population are shown in Table 1. The study groups were similar with regard to sex. Among *P*. *vivax*-infected individuals, asymptomatic patients were older than symptomatic patients and those coinfected with HBV (median age: 43yrs, IQR: 34–52 vs. 29yrs, IQR: 19–42 vs. 31yrs, IQR: 23-46yrs, respectively) (Table 1). In addition, asymptomatic malaria patients were older than healthy endemic controls but with similar median age than those with HBV monoinfection (Table 1). Of note, referred number of previous malaria episodes was lower in patients presenting with symptomatic malaria compared to those with asymptomatic malaria, malaria-HBV coinfection and those with HBV monoinfection (Table 1).

Individuals with symptomatic *P*. *vivax* infection more frequently referred that they had lived for shorter time in the malaria endemic area when compared with the other clinical groups (Table 1). As expected according to previous reports [11], parasitemia, assessed in thick blood smears, was substantially lower in individuals with HBV-malaria comorbidity compared to those with symptomatic *P*. *vivax* monoinfection (median: 753 parasites/μL, IQR: 444.3–4,262 vs. 6,324, IQR: 913.5–60,623, P = 0.0004), whereas asymptomatic malaria patients predominantly did not exhibit detectable number of parasites in peripheral blood using microscopic examination (Table 1). In addition, frequency of *P*. *vivax*-HBV coinfection was significantly higher in asymptomatic individuals (n = 25). Overall, 18 (13.04%) asymptomatic and 38 (21.23%) symptomatic vivax malaria patients presented serological status compatible with previous history of HBV infections (HBSAg-, anti-HBS+, anti-HBc+). Furthermore, all cases of HBV infection, with or without malaria co-infection, presented serological status of chronic infection. Among the 179 symptomatic vivax malaria patients, eighteen presented severe/complicated vivax malaria, and six individuals eventually died from the disease. Detailed information on symptoms presented by individuals from each clinical group is available in S1 Table.

### *P*. *vivax* malaria patients coinfected with HBV present a distinct inflammatory profile

**Fig 1:**
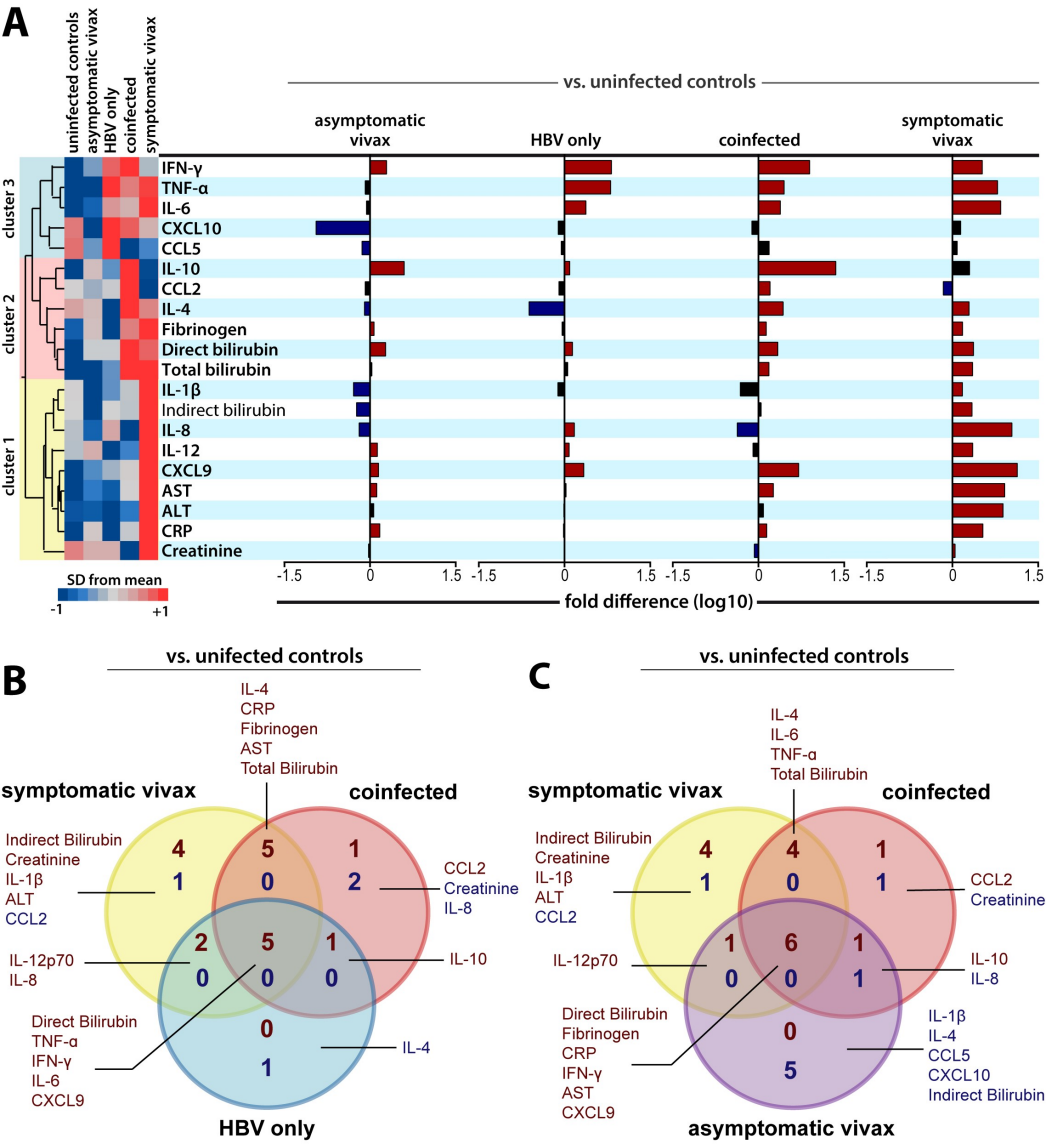
Inflammatory profile and biochemical-based distribution of HBV and *P*. *vivax* patients. (**A**) Overall profile of plasma concentrations of the biochemical parameters in uninfected controls (n = 165), asymptomatic vivax malaria subjects (n = 127), symptomatic vivax malaria patients (n = 141), HBV patients (n = 29) and *P*. *vivax*-HBV coinfected patients (n = 28). Data were processed using hierarchical cluster analysis (Ward’s method) with 100x bootstrap. Dendrograms represent hierarchical distance. The three identifiable clusters are presented and colored. Histograms represent differences of variables for each disease group alone compared to healthy control subjects using the Mann-Whitney *U* Test. Significant differences (P value < 0.05) were represented in colored bars. (**B**) Pattern of variables expressed differentially between symptomatic vivax malaria, HBV, and coinfected patients against uninfected controls. Venn’s diagram shows the number of parameters in common to two or three disease presentations, or unique to each one. (**C**) Pattern of variables expressed differentially between asymptomatic, symptomatic vivax malaria, and coinfected patients against uninfected controls. Venn’s diagram shows the number of parameters in common to two or three disease presentations, or unique to each one. Overall, the dark red color represents variables with significant elevations in the comparisons of its levels, while dark blue color represented significant reductions in the circulating concentrations of the assessed biomarkers.

Median values of all biochemical markers per group were log-transformed and z-score normalized for hierarchical cluster analysis. Using this approach, three clusters of markers were identified (Fig 1A). Fold differences of the circulating levels of all biomarkers were then calculated to assess which parameters were differentially expressed in all four main subpopulations against healthy controls (Fig 1A). Asymptomatic vivax malaria patients presented a similar number of variables with significant concentrations increases (mainly IFN-γ, IL-10 and direct bilirubin) and decreases (such as CXCL10, IL-1β, IL-4 and indirect bilirubin). Patients with HBV monoinfection presented the same number of variables which concentrations were increased (mainly IFN-γ, TNF-α, IL-6 and CXCL9), when compared to asymptomatic vivax malaria monoinfection, but only significant decrease of one variable (also IL-4). Furthermore, symptomatic vivax patients presented augmented levels of almost every analyzed analyte, except for IL-10, CCL5, CXCL10 and specifically CCL2, which levels were diminished in comparison to uninfected controls (Fig 1A). *P*. *vivax*-HBV coinfected individuals presented multiple significant elevations in biomarker values when compared to healthy controls, although not as many as found in symptomatic malaria monoinfection. Noteworthy, coinfected patients exhibited an impressive 22-fold elevation in IL-10 levels when compared to healthy controls, and remarkable decreases in IL-8 concentrations (Fig 1A). Other significant differences are illustrated in Fig 1A. Fig 1B and 1C show Venn’s diagrams to further illustrate and depict these differences and similarities initially demonstrated by the subpopulations. These results delineate the systemic inflammatory profile associated with this comorbid condition. Detailed information of the laboratorial results and analysis in the subpopulations are available in Table 2.

**Fig 2:**
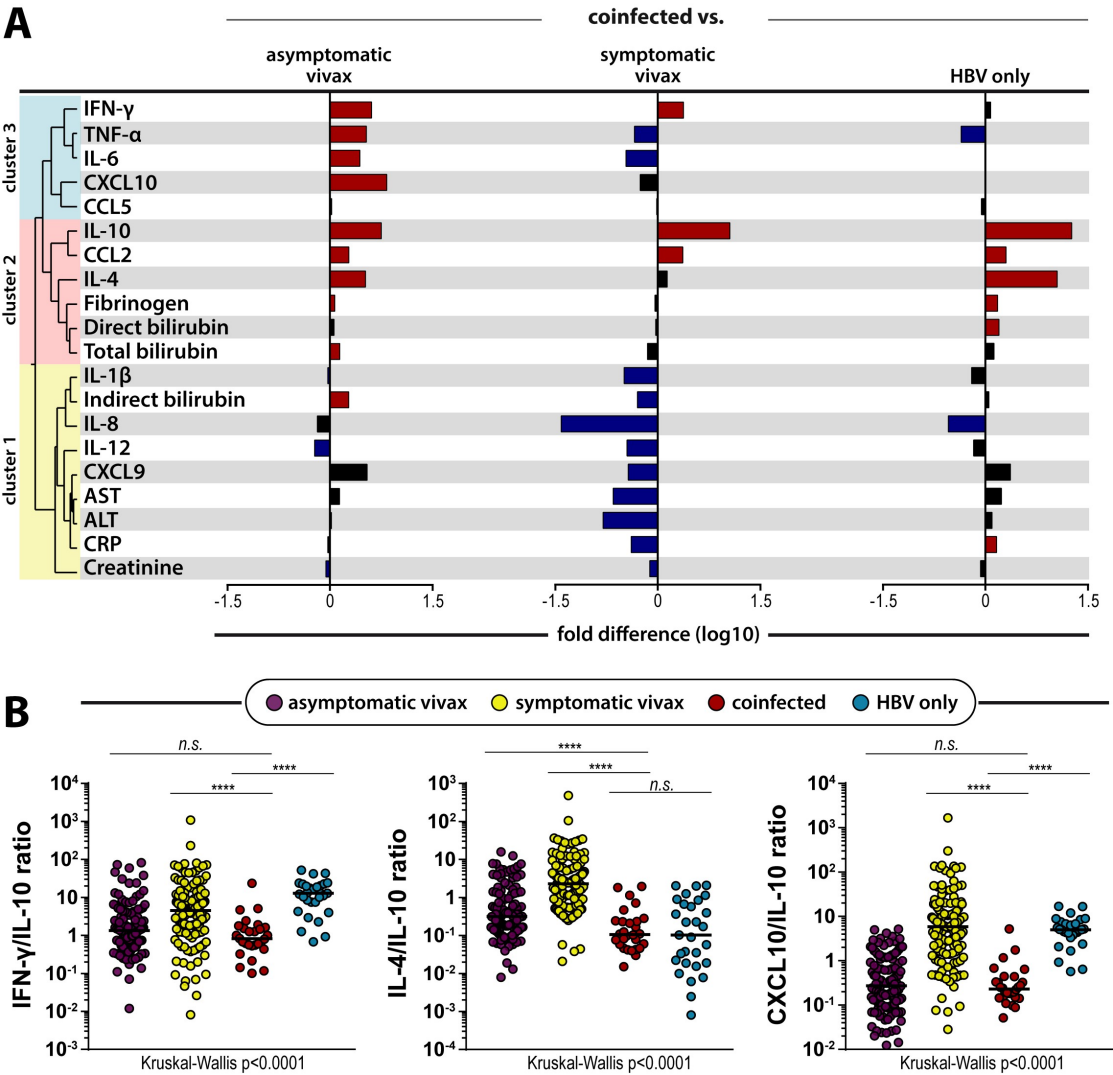
Effect of coinfection in different biomarker concentrations and its associations to monoinfected individuals. (**A**) Pattern of variables expressed differentially between symptomatic vivax malaria, and HBV patients against coinfected individuals. Dendrograms represent hierarchical distance observed from Fig 1A, and its respective clusters. (**B**) Scatter-plots of the IL-4/IL-10, CXCL10/IL-10, and the IFN-γ/IL-10 ratio, which has been shown to accurately depict the inflammatory imbalance in severe vivax malaria [6].

Fold differences of the circulating levels of all biomarkers were also calculated to assess which parameters were differentially expressed in coinfected individuals in comparison to other main study groups (coinfected *vs*. asymptomatic or symptomatic *P*. *vivax* monoinfected patients, and HBV monoinfected individuals). Patients with malaria-HBV coinfection presented elevated concentrations of multiple variables such as IFN-γ, TNF-α, IL-4, IL-10, CCL2, and reduced levels of IL-12 and creatinine levels when compared to those with asymptomatic vivax malaria monoinfection (Fig 2A). When compared to symptomatic vivax malaria patients (Fig 2A,), coinfected individuals presented elevated levels of IFN-γ, IL-10 and CCL2, and diminished plasma concentrations of multiple variables as TNF-α, IL-6, IL-12, and CRP. When compared to those with HBV monoinfection, coinfected patients presented elevated levels of IL-4, IL-10, CCL2, CRP, fibrinogen, and direct bilirubin, and reduced concentrations of TNF-α and IL-8 (Fig 2A). Other significant differences are illustrated in Fig 2A.

Thus, in summary, IL-10 and CCL2 were the only variables which coinfected patients presented with elevated concentrations in comparison to all the other three main study groups. Then, considering the immunoregulatory nature of IL-10 and the dimension of its elevations in coinfected individuals, the next step was to analyze the behavior of the biomarkers in comparison to IL-10 levels in all main study groups. Coinfected individuals presented reduced IL-10 ratios for all variables (S1 Fig), with the exception of IFN-γ, IL-4 and CXCL10 (Fig 2B). The IFN-γ/IL-10 and CXCL10/IL-10 ratios could not distinguish coinfected and asymptomatic vivax patients. In addition, HBV-monoinfected and *P*.*vivax*-HBV coinfected patients could not be distinguished by their IL-4/IL-10 ratio values. These results highlight similar biosignatures that may be reminiscent from each respective infection in *P*.*vivax*-HBV coinfected individuals.

### HBV infection leads to a shift in cytokines profile in vivax malaria patients

**Fig 3:**
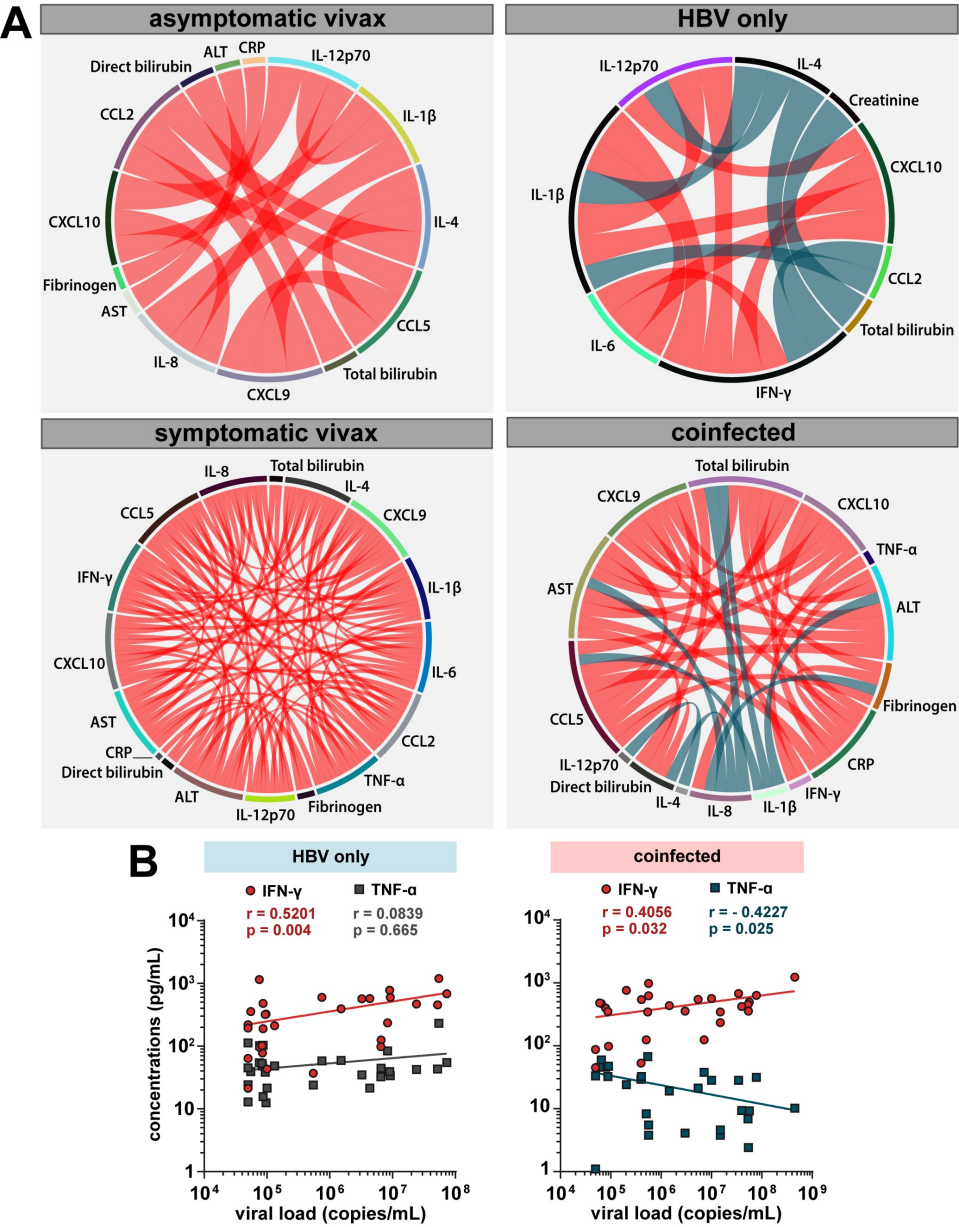
Networks of biochemical parameters during chronic HBV and vivax malaria infections. (**A**) Correlation matrices of plasma levels from multiple biomarkers of inflammation and tissue damage were evaluated in 127 asymptomatic vivax malaria, 29 HBV, 141 symptomatic vivax, and 28 coinfected patients. The colors represent whether the correlation was positive or negative in Spearman’s test (red illustrates positive correlations, blue illustrates negative correlations). Each stroke represents a significant (P<0.05) and strong (modular r value ≥ 0.6) interaction detected by the network analysis. (**B**) Correlations of IFN-γ and TNF-α levels with viral load in HBV (*upper panel*) or coinfected (*lower panel*) patients.

Multiple correlation matrices were inputted into a network analysis to assess the profile of associations between cytokine levels in each study subpopulation (Fig 3A). It was noticeable the decreased number of significant connections (which represent statistically significant correlations) in the network of asymptomatic vivax patients (Fig 3A) when compared to the networks calculated from the other groups. This tendency is also maintained when such networks were compared to that from uninfected controls (S2 Fig). In addition, the correlation matrix of individuals with symptomatic *P*. *vivax* monoinfection showed an increase of significant positive connections between the biochemical parameters (Fig 3A). Interestingly, while displaying an increased number of significant positive correlations between variables, *P*. *vivax*-HBV coinfected individuals (Fig 3A) also exhibited the tendency of negative connections presented by HBV-monoinfected patients (Fig 3A). HBV patients presented negative correlations between IL-4 and IFN-γ, IL-1β, and IL-12p70 concentrations, between CCL2 and IFN-γ or IL-1β levels, and between total bilirubin and creatinine levels. In coinfected individuals, IL-4 and IL-12p70 levels were again negatively correlated, but also between IL-8 and AST, fibrinogen, direct bilirubin, and total bilirubin concentrations. In addition, IFN-γ was only positively correlated with CXCL9 and CXCL10, whereas IL-1β levels were negatively correlated with concentrations of CCL5, ALT and total bilirubin (Fig 3A).

The further step was to analyze the correlations between the biomarkers and viral load, which has been previously associated with a downregulated proinflammatory response in individuals chronically infected with HBV [14,15]. Overall, concentrations of ten biomarkers were significantly correlated with viremia levels in patients with HBV monoinfection or in those with HBV-malarial coinfection. Furthermore, TNF-α did not presented the same correlation pattern in the two groups, which was the case of IFN-γ concentrations, for example (Fig 3B). In HBV-monoinfected individuals, TNF-α levels was not correlated with the viral load, while presenting negative correlation with viremia in coinfected patients. The other significant correlations to viral load are shown in S2 Fig.

### Coinfected individuals present a distinct inflammatory profile when compared to *P*. *vivax* monoinfected subjects, independently of clinical presentation

We next performed additional analyses in which symptomatic and asymptomatic *P*. *vivax* monoinfected subjects were considered as a single group (S3 Fig). When compared with uninfected controls, monoinfected *P*. *vivax* individuals presented the characteristic significant reduction of CXCL10 and CCL2 levels and increases of IL-10 and TNF-α levels (S3A Fig) found in previously separated groups (Fig 1A). In addition, when compared against coinfected patients, monoinfected *P*. *vivax* individuals presented significant elevations of AST, ALT, CRP, IL-8 and IL-12 (S3A Fig), and significant reductions in IFN-γ, CXCL10, CCL2, IL-4 and IL-10 (S3A Fig). The IFN-γ/IL-10 and CXCL10/IL-10 ratios could distinguish coinfected patients from both groups of *P*. *vivax* and HBV-monoinfected patients (S3B Fig). S3C Fig shows the distribution of the patients based on their parasitemia values. When compared with those presenting HBV coinfection, *P*. *vivax*-monoinfected patients presented with significantly reduced parasite counts (S3D Fig).

## Discussion

In the present study, we performed novel analyses of multiple inflammatory biomarkers related to key immune and inflammatory responses associated with disease progression in the context of HBV and *Plasmodium vivax* infections. These expanded analyses provide deeper comprehension of the immune response against *P*. *vivax*-HBV coinfection, which culminates with reduced odds of severe disease and progression of vivax malaria [11].

In the study population, asymptomatic vivax and coinfected patients presented distinct epidemiological profiles. Elevated number of previous malaria episodes and more advanced age are well-known to be associated with milder and asymptomatic vivax malaria [6,11,31]. However, while referring a similarly increased number of previous malaria episodes, coinfected patients were predominantly younger than asymptomatic vivax patients (Table 1). Furthermore, coinfected individuals presented similar median age to symptomatic *P*. *vivax* malaria patients (Table 1). Moreover, asymptomatic monoinfected vivax patients predominantly presented with undetectable parasitemia examined by thick smears, whereas coinfected individuals, predominantly asymptomatic from a malarial perspective, presented an elevated values of parasite counts (S3C Fig). In fact, coinfected individuals presented significantly increased parasite counts when compared to *P*. *vivax* monoinfection (S3D Fig). Hence, these distinct epidemiological and serological characteristics of asymptomatic vivax and coinfected patients argues that other factors may have influenced malarial presentations in coinfection with HBV. Moreover, the parasitemia results are in line with previous hypothesis that mainly the host response to infection, and not the parasite load alone, are responsible for clinical presentations in vivax malaria [5,6,22–24]. Multiple cytokines, chemokines and acute phase proteins were then profiled to further analyze the mechanisms associated with disease presentation in *P*. *vivax*-HBV coinfected patients. Coinfection was hallmarked by extremely elevated concentrations of IL-10, as well as heightened levels of CCL2, in comparison to the distinct clinical presentations of *P*. *vivax* infections or HBV monoinfection.

IL-10 is an immunoregulatory cytokine and its levels were previously reported to be closely associated with disease progression and outcomes in both hepatitis B and vivax malaria. Patients with severe vivax malaria have been shown to present unbalanced concentrations of IL-10 against levels of proinflammatory biomarkers, when compared to individuals with uncomplicated vivax malaria [5,6,22]. In addition, these individuals with uncomplicated or asymptomatic *P*. *vivax* infections have been shown to express relatively augmented concentrations of IL-10 when compared to those with symptomatic or severe vivax malaria [5,6,31,32]. In viral infections, IL-10 levels are associated with diminished T-cell activation, which may already start to occur rapidly after infection [20]. Furthermore, HBV actively suppresses immune responses [14] and augmented IL-10 levels are closely associated with viral persistence [16–18,33]. Herein, as expected, HBV-infected patients presented increased IL-10 levels when compared to uninfected controls. Furthermore, only symptomatic vivax malaria patients could not be distinguished from uninfected controls by their IL-10 levels. Although coinfected patients presented almost an 8-fold increase in IL-10 concentrations when compared to asymptomatic vivax-monoinfected individuals, they presented undistinguishable values of IFN-γ/IL-10 ratios, which highlights a similar tendency of immune balance in this aspect of inflammatory response. A higher baseline concentration of IL-10, associated with the condition of antiviral response, and hence a distinct overall inflammatory profile, could then be responsible for the difference in absolute IL-10 levels identified between coinfected and asymptomatic vivax patients. Both study groups also presented similar CXCL10/IL-10 ratio values. CXCL10 is an IFN-γ induced protein which acts in chemotaxis, apoptosis, cell proliferation and angiogenesis [34]. Thus, similar results of CXCL10/IL-10 and IFN-γ/IL-10 are not surprising. Even with these solid findings, experimental models are still necessary to further define and ratify whether these similarities were directly carried from responses associated to HBV persistence and T-cell exhaustion, or from the antimalarial response, or from concurrent responses to both pathogens.

CCL2, the other biomarker which concentrations are augmented in coinfected individuals in comparison to all other study groups, is an important chemokine involved in recruitment of monocytes [35,36] and NK cells [36]. CCL2 is reported to be produced by hepatocytes under acute HBV stress [37] and was previously associated with uncomplicated *P*. *vivax* infections [31]. However, without the weight of an acute and heavy antiviral response, an infection with a pathogen known to induce acute hepatocyte damage as *P*. *vivax* [6] could possibly trigger this local chemokine production. ALT and AST levels could reinforce this hypothesis as they were found to be augmented in symptomatic vivax malaria patients when compared to HBV-monoinfected individuals (Table 2), while only being correlated to viremia in coinfected *P*. *vivax*-HBV patients and not in HBV-monoinfected subjects (S2 Fig). Herein, coinfected individuals, which presented predominantly asymptomatic malarial infection, had a 2.2-fold increase in CCL2 concentrations when compared to patients with symptomatic *P*. *vivax* infections. Therefore, these results may reinforce the association of CCL2 with uncomplicated malaria.

It is also reported that CCL2 influences and directs CD4^+^ T lymphocytes to a more biased response towards IL-4 production [38]. Herein, coinfected individuals presented significant elevations IL-4 levels when compared to healthy controls (Fig 1A), asymptomatic vivax or HBV-monoinfected subjects (Fig 2A). Although IL-4 levels could not distinguish coinfected and symptomatic vivax malaria patients, correlations with IL-4 concentrations were completely different in both study groups. IL-4 concentrations were negatively correlated to IL-12p70 levels in coinfected patients, while being positively correlated with multiple other proinflammatory cytokines in symptomatic vivax patients (Fig 3A). Hence, these antagonic tendencies suggests that different mechanisms, and not just the antimalarial response in this case, could be responsible for the elevation of IL-4 concentrations in coinfected and symptomatic vivax malaria patients. In addition, IL-4/IL-10 ratio values could not distinguish coinfected and HBV-monoinfected individuals (Fig 2B). This similar profile displayed in both groups of patients infected by HBV suggests that antiviral or responses associated with hepatocyte stress, possibly under CCL2 influence in this hypothesis, could be responsible for these elevations of IL-4 levels in coinfected individuals. In practical terms, this immune response of coinfected individuals with augmented IL-4 concentrations happens without much proinflammatory pressure, as IL-10 immunoregulatory mechanisms should be expected to bring them a more balanced inflammatory response, oppositely to what occurs in symptomatic vivax individuals (Fig 1A). This augmented production of IL-10 alongside IL-4 heightened levels can directly downregulate key proinflammatory cytokines such as TNF-α [39,40], and thus have a protective effect against severe malaria presentations. Herein, TNF-α concentrations were found to be significantly reduced in coinfected individuals, when compared to both HBV or symptomatic vivax malaria patients (Fig 2A). Furthermore, TNF-α concentrations were negatively correlated to viremia only in patients with *P*. *vivax*-HBV coinfections (Fig 3B), which could be read as a possible effect of these previously reported mechanisms in the patients from the present study. These results are compatible with the hypothesis that coinfection drives reduction of systemic inflammation, which we previously published [11]. Therefore, these confluent events from responses to both pathogens (increased production of IL-10, CCL2 protective role in malaria, as well as combined effects of IL-4 and IL-10) could enable the host to respond properly without unbalanced inflammation. Thus, this proper response creates an environment unfavorable for the *Plasmodium* to thrive and induce detectable symptoms.

Our study presented some limitations. We did not have data available from follow-up of the HBV-infected patients and their antiviral treatments, as they were referred to a specialized service. Although we collected information regarding number of previous malaria episodes, these data were expressed by the patients, and not extracted from official documents of the health centers. Thus, this fact further limits the analysis and evaluation of relapses in the study patients, and if these events could be related to an association between the chronic HBV infection and hypnozoite activation. Experimental models and biopsies would have helped with the assessment of T-cell exhaustion, the impact of liver involvement into inflammatory responses, and cytokine evaluation at tissue level. Therefore, further longitudinal and experimental studies are still necessary to completely understand the events associated with *P*. *vivax*-HBV coinfection. However, despite some limitations, the present study was successful to analyze several biomarkers and their associated biomechanisms, and link them to the known protective effect of chronic HBV infections in vivax malaria.

In conclusion, the results presented here represent a translation of an increased demand and pressure caused by the acute *P*. *vivax* infection on the immune system of a chronically HBV-infected host. Hence, there is an augmented presence of inflammatory biomarkers as IFN-γ and CRP, counterbalanced with the immunoregulatory mechanisms discussed here. In summary, coinfection was hallmarked by substantially increased levels of IL-10 and augmented concentrations of CCL2. CCL2 is expressed by hepatocytes during acute injury, reportedly leads to IL-4 increases, while IL-10 is directly related to viral persistence and T-cell exhaustion, and both cytokines are associated with protection in *P*. *vivax* infections. Thus, these results argue that distinct mechanisms associated with antiviral and antimalarial activity are due to changes in cytokine balance, and lead to the known increased odds of asymptomatic vivax malaria in coinfected HBV-*P*. *vivax* patients. This knowledge of responses to both pathogens counteracting proinflammatory responses helps to depict the pathophysiology associated with the coinfection, and could prove relevant to future studies and approaches with immunotherapy in cases of severe malaria or HBV infection.

## Supporting information

S1 Table Clinical characteristics of the study participants. Data were compared using the exact Fisher’s test or the chi-square test. P value 1 refers to comparisons of data from all the represented subgroups. P value 2 refers to comparisons between symptomatic *P*. *vivax* monoinfected and HBV-*P*. *vivax* coinfected patients. P value 3 refers to comparisons between HBV monoinfected and HBV-*P*. *vivax* coinfected patients. *178 symptomatic *P*. *vivax* malaria had information for the symptoms available. **177 symptomatic *P*. *vivax* malaria patients had information for the symptoms available.
(PDF)

S1 Fig IL-10 ratios inflammatory profiling. Scatter-plots representing IL-10 ratios for each chemokine and cytokine, bar IL-4, CXCXL10 and IFN-γ, which are represented in Fig 2B.
(TIF)

S2 Fig Control group correlation matrix and viremia correlations to inflammatory biomarkers. (**A**) Reference correlation matrix of the uninfected control group. The colors represent whether the correlation was positive or negative in Spearman’s test) red illustrates positive correlations, blue illustrates negative correlations). Each stroke represents a significant (P<0.05) and strong (modular r value ≥ 0.6) interaction detected by the network analysis. (**B**) Inflammatory biomarkers correlated to viremia in HBV or HBV-*P*. *vivax* coinfected patients.
(TIF)

S3 Fig Differently expressed markers when asymptomatic and symptomatic P. vivax-monoinfected patients are considered as one single group. (**A**) Pattern of variables expressed differentially between *P*. *vivax* monoinfection and uninfected controls, or coinfected patients. Significant increases and decreases in the concentrations for each variable are shown in orange and purple, respectively. (**B**) Scatter-plots of the CXCL10/IL-10 ratio and the IFN-γ/IL-10 ratio, which has been shown to accurately depict the inflammatory imbalance in severe vivax malaria [6]. (**C**) Histogram representing parasitemia of all the *P*. vivax monoinfected patients (upper panel) and of coinfected patients (lower panel). Each bar represents one patient. Patients are colored accordingly to the type of infection or disease presentation. (**D**) Scatter-plot of the parasitemia presented by the subpopulations of *P*. *vivax* monoinfected patients overall and coinfected subjects. Data analysis was performed using the Mann-Whitney *U* test. Bars represent median values.
(TIF)
