# Supplementary material for: Chronic hepatitis B virus infection drives changes in systemic immune activation profile in patients coinfected with Plasmodium vivax malaria
Source: PLoS Negl Trop Dis. 2019 Jun 24;13(6):e0007535. doi: 10.1371/journal.pntd.0007535 (PMC6611654; doi:10.1371/journal.pntd.0007535)
Supplement: S1 Table — Data were compared using the exact Fisher’s test or the chi-square test. P value 1 refers to comparisons of data from all the represented subgroups. P value 2 refers to comparisons between symptomatic P. vivax monoinfected and HBV-P. vivax coinfected patients. P value 3 refers to comparisons between HBV monoinfected and HBV-P. vivax coinfected patients. *178 symptomatic P. vivax malaria had information for the symptoms available. **177 symptomatic P. vivax malaria patients had information for the symptoms available. (PDF) [file pntd.0007535.s001.pdf]

S1 Table: Clinical characteristics of the study participants

Variables	*P. vivax* malaria patients		HBV only	*symptomatic vivax vs. coinfected vs. HBV only* **P value 1**	*symptomatic vivax vs. coinfected* **P value 2**	*HBV only vs. coinfected* **P value 3**
	**symptomatic**	**HBV coinfected**				
**n**	179	28	29			
**Fever**	179 (100.0)	3 (10.71)	0 (0.00)	< 0,0001	< 0,0001	0,1120
**Chill**	148 (82.68)	5 (17.85)	0 (0.00)	< 0,0001	< 0,0001	0,0235
**Sweating**	110* (61.79)	8 (28.57)	0 (0.00)	< 0,0001	0,0016	0,0019
**Headache**	100 (55.86)	8 (28.57)	6 (20.69)	0,0002	0,0082	0,5497
**Myalgia**	95 (53.07)	5(17.85)	0 (0.00)	< 0,0001	0,0005	0,0235
**Arthralgia**	98** (55.36)	5 (17.85)	0 (0.00)	< 0,0001	0,0002	0,0235
**Low back pain**	64 (35.75)	0 (0.00)	0 (0.00)	< 0,0001	< 0,0001	-
**Nausea**	106 (59.21)	0 (0.00)	0 (0.00)	< 0,0001	< 0,0001	-
**Vomiting**	22 (12.29)	0 (0.00)	0 (0.00)	0,0210	0,0497	-
**Hyporexia**	70 (39.10)	0 (0.00)	0 (0.00)	< 0,0001	< 0,0001	-
**Bleeding**	0 (0.00)	0 (0.00)	0 (0.00)	-	-	-
**Disorientation**	22* (12.35)	0 (0.00)	0 (0.00)	0,0205	0,0495	-
**Jaundice**	31 (17.31)	0 (0.00)	0 (0.00)	0,0034	0,0102	-
**Choluria**	8 (4.46)	0 (0.00)	0 (0.00)	0,2676	0,6016	-
**Hypocholia/acholia**	4** (2.25)	0 (0.00)	0 (0.00)	0,5193	1,0000	-
**Weakness**	163 (91.06)	0 (0.00)	13 (30.95)	< 0,0001	< 0,0001	< 0,0001
**Palpitations**	28 (15.64)	0 (0.00)	0 (0.00)	0,0064	0,0175	-
**Dizziness**	29 (16.20)	0 (0.00)	0 (0.00)	0,0052	0,0171	-
**Abdominal pain**	12 (6.70)	0 (0.00)	0 (0.00)	0,1336	0,3764	-
**Diarrhea**	22 (12.29)	0 (0.00)	8 (27.58)	0,0071	0,0497	0,0045
**Pallor**	55 (30.72)	2 (7.14)	10 (34.48)	0,0269	0,0108	0,0206
**Seizures**	10 (5.58)	0 (0.00)	0 (0.00)	0,1896	0,3641	-
**Bad General Condition**	11 (6.14)	0 (0.00)	0 (0.00)	0,1593	0,3669	-
**Collateral Circulation**	0 (0.00)	0 (0.00)	2 (6.89)	0,0007	-	0,4912
**Bleached mucosa**	67 (37.43)	0 (0.00)	2 (6.89)	< 0,0001	< 0,0001	0,4912
**Dehydration**	30 (16.75)	0 (0.00)	2 (6.89)	0,0294	0,0174	0,4912
**Petechiae**	12 (6.70)	0 (0.00)	0 (0.00)	0,1336	0,3764	-
**Skin Rash**	8 (4.46)	0 (0.00)	0 (0.00)	0,2676	0,6016	-
**Tachycardia**	41 (22.90)	0 (0.00)	2 (6.89)	0,0034	0,0018	0,4912
**Tachypnea**	13 (7.26)	0 (0.00)	0 (0.00)	0,1119	0,2229	-
**Hepatomegaly**	23 (29.11)	3 (10.71)	5 (17.24)	0,7463	1,0000	0,7057
**Splenomegaly**	13 (7.26)	0 (0.00)	0 (0.00)	0,1119	0,2229	-

Data were compared using the exact Fisher's test or the chi-square test. P value 1 refers to comparisons of data from all the represented subgroups. P value 2 refers to comparisons between symptomatic *P. vivax* monoinfected and HBV-*P. vivax* coinfected patients. P value 3 refers to comparisons between HBV monoinfected and HBV-*P. vivax* coinfected patients. *178 symptomatic *P. vivax* malaria had information for the symptoms available. **177 symptomatic *P. vivax* malaria patients had information for the symptoms available.
