# Supplementary figures and images for: Chronic hepatitis B virus infection drives changes in systemic immune activation profile in patients coinfected with Plasmodium vivax malaria
Source: PLoS Negl Trop Dis. 2019 Jun 24;13(6):e0007535. doi: 10.1371/journal.pntd.0007535 (PMC6611654; doi:10.1371/journal.pntd.0007535)

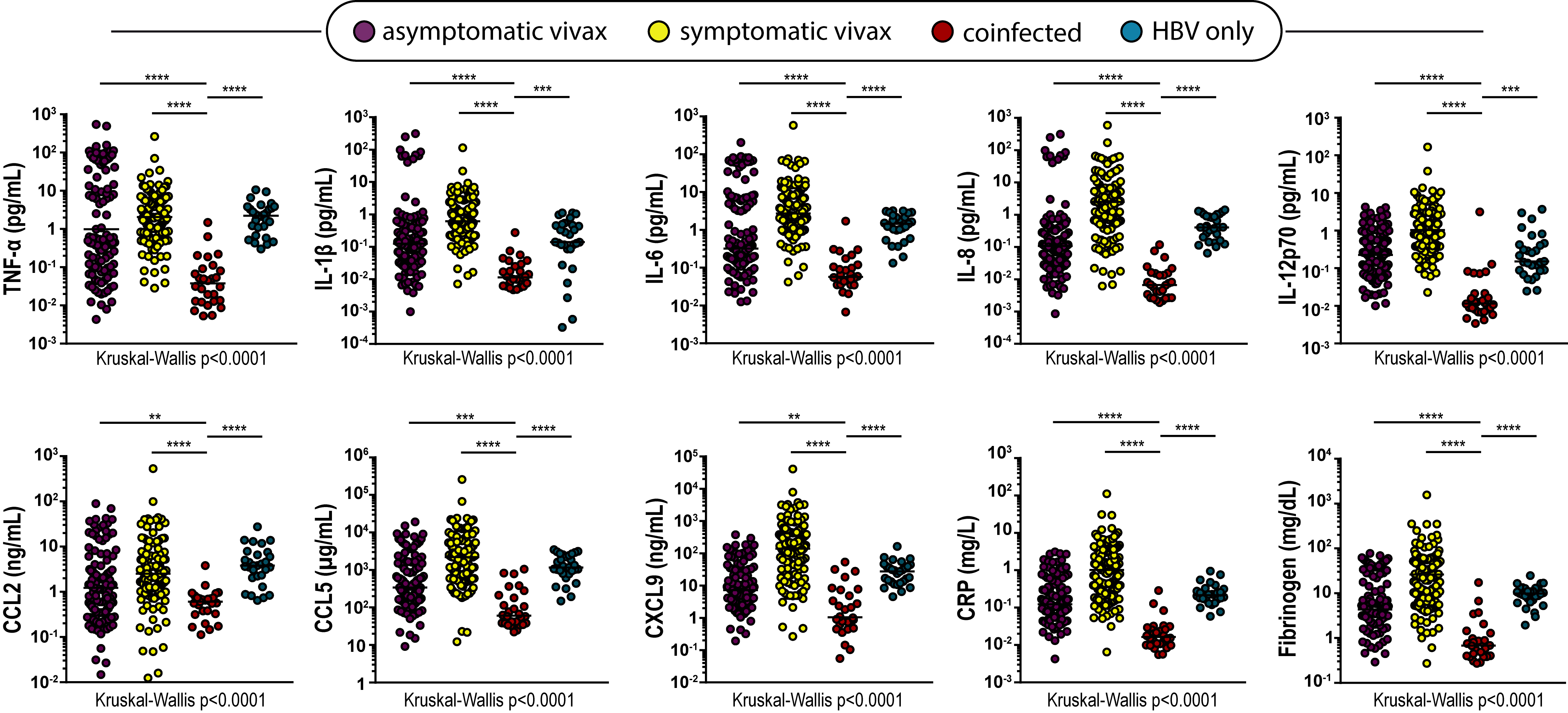

Supplement: S1 Fig — Scatter-plots representing IL-10 ratios for each chemokine and cytokine, bar IL-4, CXCXL10 and IFN-γ, which are represented in Fig 2B. (TIF) [file pntd.0007535.s002.tif]

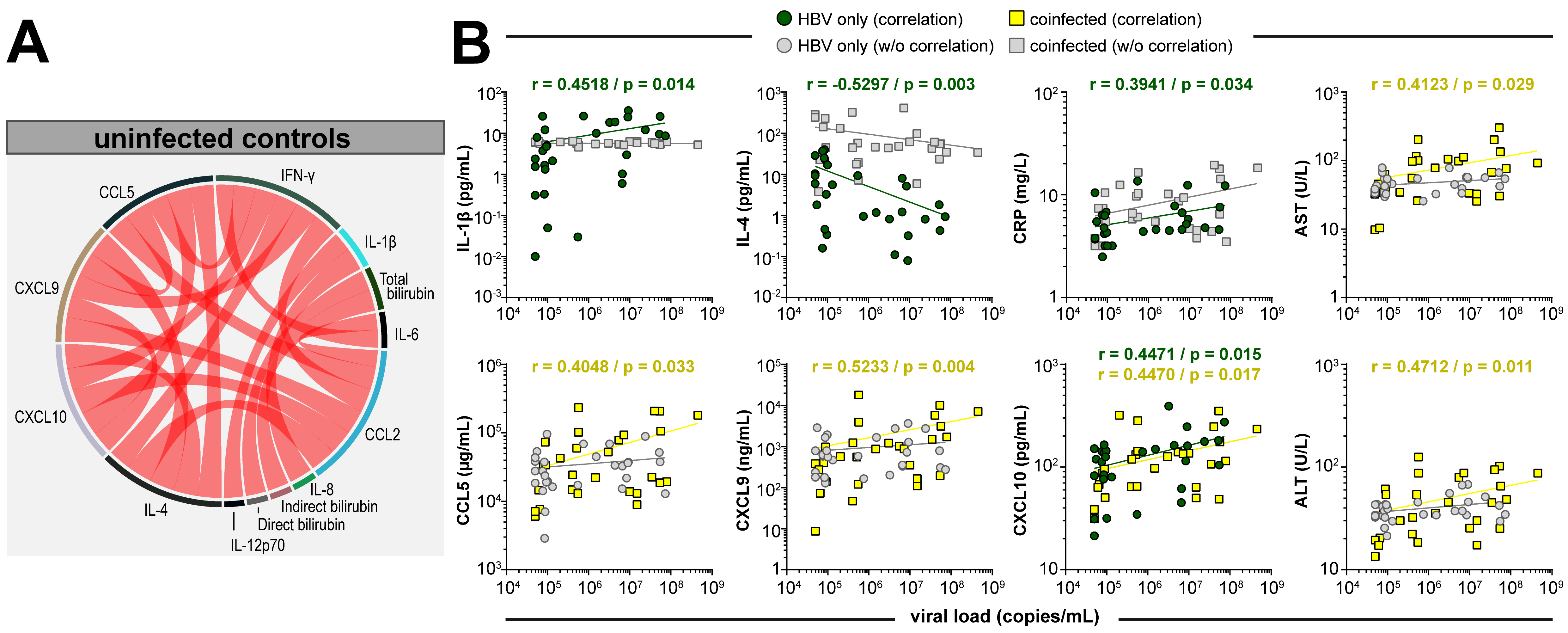

Supplement: S2 Fig — (A) Reference correlation matrix of the uninfected control group. The colors represent whether the correlation was positive or negative in Spearman’s test) red illustrates positive correlations, blue illustrates negative correlations). Each stroke represents a significant (P<0.05) and strong (modular r value ≥ 0.6) interaction detected by the network analysis. (B) Inflammatory biomarkers correlated to viremia in HBV or HBV-P. vivax coinfected patients. (TIF) [file pntd.0007535.s003.tif]

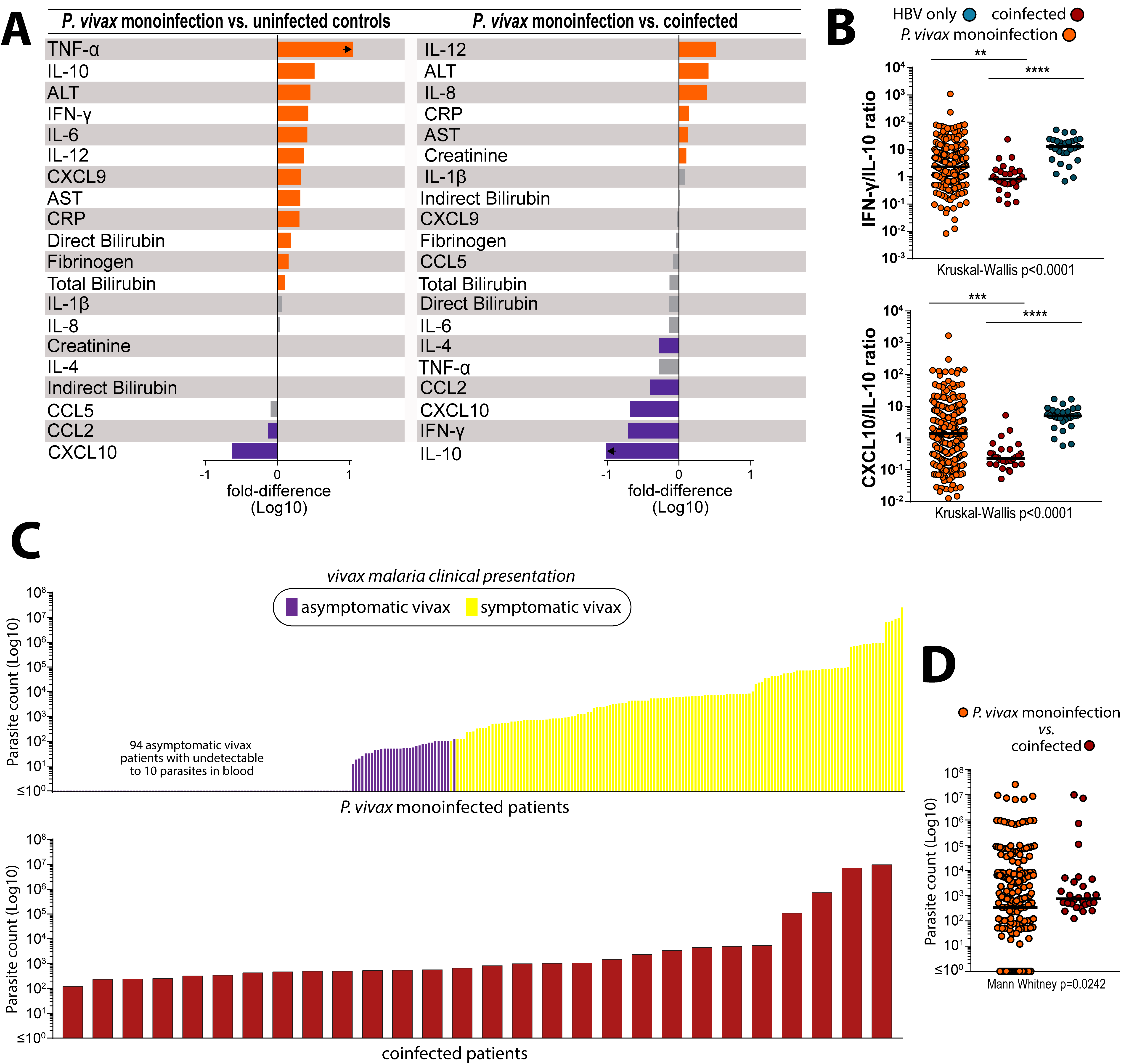

Supplement: S3 Fig — (A) Pattern of variables expressed differentially between P. vivax monoinfection and uninfected controls, or coinfected patients. Significant increases and decreases in the concentrations for each variable are shown in orange and purple, respectively. (B) Scatter-plots of the CXCL10/IL-10 ratio and the IFN-γ/IL-10 ratio, which has been shown to accurately depict the inflammatory imbalance in severe vivax malaria [6]. (C) Histogram representing parasitemia of all the P. vivax monoinfected patients (upper panel) and of coinfected patients (lower panel). Each bar represents one patient. Patients are colored accordingly to the type of infection or disease presentation. (D) Scatter-plot of the parasitemia presented by the subpopulations of P. vivax monoinfected patients overall and coinfected subjects. Data analysis was performed using the Mann-Whitney U test. Bars represent median values. (TIF) [file pntd.0007535.s004.tif]
